# Association between niacin intake and chronic kidney disease in male participants—a cross-sectional study from the NHANES (2005–2018)

**DOI:** 10.3389/fnut.2025.1578118

**Published:** 2025-06-13

**Authors:** Cong Hu, Ting Tang

**Affiliations:** Department of Traditional Chinese Medicine, The Central Hospital of Wuhan, Tongji Medical College, Huazhong University of Science and Technology, Wuhan, Hubei, China

**Keywords:** niacin intake, chronic kidney disease, NHANES, restricted cubic splines, cross-sectional study

## Abstract

**Background:**

Chronic kidney disease (CKD), a significant health challenge in the United States, often progresses from asymptomatic conditions to advanced stages and exhibits a higher prevalence among male individuals. Niacin is known for its metabolic and antioxidant roles, potentially influencing CKD progression. The association between niacin intake and CKD has been rarely investigated in male participants.

**Methods:**

This cross-sectional study utilized data of 13,946 male participants aged above 18 years from the National Health and Nutrition Examination Survey (NHANES) 2005–2018. CKD was diagnosed by albumin-to-creatinine ratio (ACR) and estimated glomerular filtration rate (eGFR). The niacin intake was recorded according to two 24-h dietary recalls. The association between niacin intake and CKD in male participants was examined using weighted logistic regression models, restricted cubic splines, and stratified analyses.

**Results:**

The prevalence of CKD was 17.07%. There was a prominent non-linear relationship between niacin intake and CKD (*p*-non-linear < 0.05). The inflection point for niacin intake was 26.79 mg (*p* for log-likelihood ratio < 0.05). The adjusted odds ratios (ORs) for the highest quartile of niacin intake and CKD were 0.752 (95% confidence interval [CI]: 0.591–0.959). In addition, race, body mass index (BMI), and cardiovascular disease (CVD) were significantly associated with this relationship (*p* for interaction < 0.05). However, poverty-to-income ratio (PIR), education level, smoking status, alcohol consumption, hypertension, and diabetes were not statistically significantly associated with the relationship between niacin intake and CKD in male patients (*p* for interaction > 0.05).

**Conclusion:**

In male patients, the niacin intake will reduce the risk of CKD.

## 1 Introduction

Chronic kidney disease (CKD) is an irreversible disease with high mortality and numerous complications, which ranks as the principal leading cause of mortality in the United States (1, 2). The overall crude prevalence of CKD increased from 12.9% between 2001 and 2004 to 13.9% between 2017 and March 2020 (3). Symptoms of CKD typically do not appear or are not noticeable in early stages, and typical complications of renal insufficiency only occur in the later stages. In addition, CKD significantly increases the risk of kidney failure, stroke, and heart attack (4). Currently, CKD is treated with dietary therapy, pharmacotherapy, dialysis, and renal transplant, among which dialysis is the treatment for the final stage. However, it can lead to hypotension and increase the risk of heart conditions (5). Therefore, prevention, screening, and treatment of CKD are crucial.

The prevalence of CKD among female participants is twice as high as that among male participants in France, Thailand, and Turkey. However, male participants account for approximately 60% of the end-stage renal disease (ESRD) population, representing a higher proportion than female participants do (6, 7). A 10-year follow-up study among Norwegian participants indicates that the risk of mortality is 1.30 times higher for male than for female participants (7). Therefore, focusing on the male population is crucial to explore the impact of CKD and improve the prognosis and outcomes for male CKD patients.

Niacin (Vitamin B3), a nutritional precursor for nicotinamide adenine dinucleotide (NAD) and NAD phosphate, is involved in cellular reactions, energy metabolism, and redox reactions (8).

A cross-sectional study revealed that daily niacin intake may play a potential role against non-alcoholic fatty liver disease (NAFLD), and participants who consumed over 22 mg of niacin had a lower risk of NAFLD than those with niacin intake of < 16.4 mg (9). Pan et al. investigated the association between niacin intake and the risk of all-cause mortality and cardiovascular disease (CVD) mortality in NAFLD individuals. They demonstrated that higher niacin intake was associated with a lower risk of all-cause mortality, but not with CVD mortality (10). The lack of niacin intake during pregnancy would elevate the risk of congenital anomalies in the offspring (11). When niacin intake is under 36 mg/day, the higher niacin intake decreases the risk of depression (12). The existing research study has shown that the OS can lead to severe mitochondrial damage, resulting in oxidative damage to renal cells (13). The energy metabolism of CKD populations is lower than normal levels (14). Therefore, OS negatively affects CKD progression as it exacerbates the condition of CKD. Nonetheless, there is a dearth of research on the direct association between niacin intake and CKD.

This study aims to explore the association between niacin intake and CKD in male participants from the National Health and Nutrition Examination Survey (NHANES).

## 2 Materials and methods

### 2.1 Study population

This study is based on data from NHANES 2005–2018. NHANES is a nationally representative survey of the US civilian non-institutionalized population. It is an ongoing survey using a probability-based sampling method to select the participants. NHANES has received approval from the Ethics Review Committee of the National Center for Health Statistics, and all participants have submitted written informed consent forms. The statistics are accessible at https://www.cdc.gov/nchs/nhanes/.

**Figure 1:**
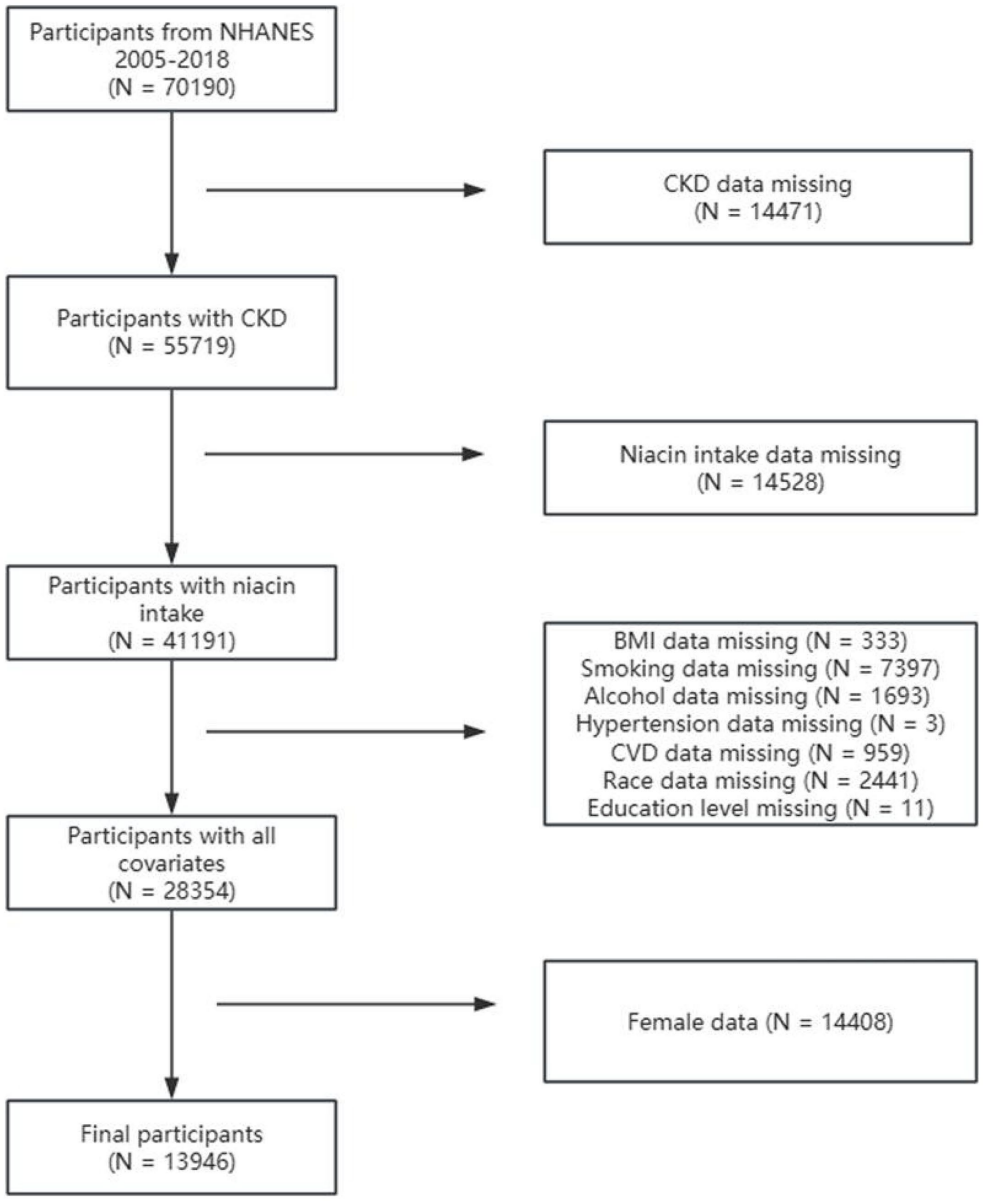
Flowchart of sample selection from NHANES 2005–2018.

Among 70,190 participants from NHANES 2005–2018, individuals were excluded for the following reasons: (1) incomplete CKD data (*N* = 14,471), (2) missing niacin intake data (*N* = 14,528), (3) missing covariate data on body mass index (BMI) (*N* = 333), smoking status (*N* = 7397), alcohol consumption (*N* = 1,693), hypertension (*N* = 3), CVD (*N* = 959), race (*N* = 2,441), and education level (*N* = 11), and (4) female sex (*N* = 14,408). Finally, this research included 13,946 participants (Figure 1).

### 2.2 CKD (outcome)

CKD was defined by albumin-to-creatinine ratio (ACR) and estimated glomerular filtration rate (eGFR). The ACR was estimated using the ratio of urine albumin to creatinine. eGFR was calculated using the CKD Epidemiology Collaboration algorithm (15):

$$\begin{array}{l} \text{eGFR}=141\times \min{\left(\frac{\mathrm{Scr}}{\kappa },1\right)}^{\alpha }\times \max{\left(\frac{\mathrm{Scr}}{\kappa },1\right)}^{-1.209}\\ \times 0.993^{\mathrm{Age}}\times 1.018\;[\text{if female}]\times 1.159\;[\text{if black}], \end{array}$$

where Scr denotes the serum creatinine concentration, *κ* is 0.7 for female participants and 0.9 for male participants, and *α* is −0.329 for female participants and −0.411 for male participants. The patients diagnosed with CKD were defined according to ACR of ≥ 30 mg/g or eGFR of ≤ 60 mL/min/1.73 m^2^.

### 2.3 Niacin intake (exposure)

NHANES collected dietary information through 24-hour recall interviews at the Mobile Examination Center (MEC). This study estimated the niacin intake of the participants through two reliable 24-hour recall intakes or one considered reliable single recall, excluding supplements and medications. Then, the intake of niacin is a continuous variable, with the unit being mg. Then, niacin intake was divided into quartiles from lowest (Q1) to highest (Q4), which are Q1: 0–21.483 mg, Q2: 21.483–28.521 mg, Q3: 28.521–37.098 mg, Q4: 37.098 mg or more.

### 2.4 Covariate

The covariates included age, race, poverty-to-income ratio (PIR), education level, smoking status, consuming alcohol, body mass index (BMI), hypertension, diabetes, and CVD. Age is a continuous variable, ranging from 20 to 85 years. The race categories included Mexican–American, other Hispanic, non-Hispanic white, non-Hispanic Black, and others. The education level was classified as under high school, high school, and college. PIR encompassed three categories: ≤1.3, 1.3–3.5, and >3.5 (16). Smoking status was categorized as non-smoker, former smoker, and current smoker. Participants who were categorized as non-smokers were categorized as having smoked <100 cigarettes in their lives. Participants who were categorized as former smokers were categorized as having smoked >100 cigarettes but were not currently smoking. Current smokere were categorized as having smoked >100 cigarettes and were still smoking. Alcohol consumption status was categorized as having at least 12 alcoholic drinks per year or not. BMI was categorized as underweight (<25 kg/m^2^), normal weight (25–30 kg/m^2^), and overweight (≥30 kg/m^2^) (17). Participants were defined as having hypertension if they (1) had been told by the doctor that they had hypertension, (2) took an antihypertensive prescription, or (3) had systolic pressure of > 130 mm Hg or diastolic pressure of >80 mm Hg. Participants were defined as having diabetes if they (1) had been informed by a healthcare provider that they had diabetes, (2) took oral diabetic pills or insulin, (3) had fasting blood glucose of >126 mg/dL, or (4) had random blood glucose of >11.1 mmol/L or glycated hemoglobin HbA1c of >6.5% (18). CVD was defined as a combination of five self-reported conditions: congestive heart failure, coronary heart disease, heart attack, angina pectoris, and stroke (19).

### 2.5 Statistical analysis

Given the complex sampling design of the NHANES dataset, appropriate weights from the first and second days of dietary data were used. All continuous variables were presented as medians, first quartiles, and third quartiles. The categorical variables were presented as frequency and weighted percentage (%). To examine differences in the characteristics between CKD and non-CKD participants, the Mann–Whitney test was used for continuous variables, while the chi-squared (*χ*^2^) test was used for categorical variables. Three different models were constructed using weighted multivariate logistic regression analysis (LRA) to determine the association between niacin intake and CKD in males. Niacin intake was analyzed as a continuous variable and as a categorical variable divided into quartiles. Trend analysis was performed using the Wald test. Model 1 was unadjusted with no covariates. Model 2 adjusted for race, age, education level, and PIR. Model 3 was further adjusted for smoking status, alcohol consumption, BMI, hypertension, diabetes, and CVD based on Model 2. The association between niacin intake and CKD in male participants was assessed using odds ratios (ORs) and 95% confidence intervals (CIs). Then, restricted cubic spline (RCS) regression was employed to examine the potential non-linear association. Finally, subgroup analyses stratified by all categorical covariates were conducted. In addition, hyperuricemia was included in the “Sensitivity analysis” section, and the primary analysis was repeated to verify the robustness of the results. All statistical analyses used RStudio (R version 4.4.0), with a *p*-value of < 0.05 implying statistical significance (R Foundation and R Core Group, New Zealand).

## 3 Results

### 3.1 Baseline characteristics

A total of 13,946 participants were enrolled, with a median age of 46 years, and 17.07% of the participants had CKD.

Baseline characteristics of CKD and non-CKD individuals are presented in Table 1. The differences in age, race, education, PIR, smoking status, alcohol consumption, BMI, hypertension, diabetes, and CVD were statistically significant (*p* < 0.05). Compared to individuals without CKD, CKD patients tended to be elderly, to be non-Hispanic, to have less than a high school, to have a PIR of < 3.5, to be former smokers, to have a BMI of ≥30 kg/m^2^, to have hypertension, and to have CVD.

**Table 1:** Participant sociodemographic characteristics in NHANES cycles: 2005–2018.

Characteristic	Total (13,946)	Non-CKD (11,566)	CKD (2,380)	*p*-value
Age (years)	46(32, 59)	43(31, 56)	64(52, 74)	<0.001
Race				0.013
Mexican–American	2,130 (9.2)	1,827 (9.4)	303 (7.4)	
Other Hispanic	1,186 (5.1)	1,001 (5.3)	185 (4.1)	
Non-Hispanic White	6,409 (69)	5,221 (68)	1,188 (70)	
Non-Hispanic Black	2,802 (9.7)	2,289 (9.5)	513 (11)	
Other races	1,419 (7.4)	1,228 (7.5)	191 (7.3)	
Education				<0.001
Under high school	3,371 (15)	2,669 (14)	702 (20)	
High school	3,378 (24)	2,803 (24)	575 (24)	
College	7,197 (61)	6,094 (62)	1,103 (57)	
PIR				<0.001
≤1.3	4,026 (20)	3,315 (19)	711 (21)	
1.3–3.5	5,318 (34)	4,302 (34)	1,016 (39)	
≥3.5	4,602 (46)	3,949 (47)	653 (40)	
Smoke				<0.001
Never	6,390 (49)	5,466 (50)	924 (40)	
Former	4,248 (29)	3,225 (27)	1,023 (43)	
Current	3,308 (22)	2,875 (23)	433 (18)	
Alcohol				0.002
No	2,121 (12)	1673 (12)	448 (16)	
Yes	11,825 (88)	9,893 (88)	1,932 (84)	
BMI (kg/m^2^)				<0.001
<25	3,724 (26)	3,222 (27)	502 (17)	
25–30	5,305 (38)	4,440 (38)	865 (35)	
≥30	4,917 (36)	3,904 (35)	1,013 (47)	
Hypertension				<0.001
No	5,944 (47)	5,514 (50)	430 (21)	
Yes	8,002 (53)	6,052 (50)	1,950 (79)	
Diabetes				<0.001
No	11,312 (86)	9,976 (90)	1,336 (61)	
Yes	2,634 (14)	1,590 (10)	1,044 (39)	
CVD				<0.001
No	12,248 (91)	10,617 (93.7)	1,631 (72)	
Yes	1,698 (9)	949 (6.3)	749 (28)	

Median (Q1, Q3) for continuous variables: the *p*-value was computed by the weighted linear regression model. Frequency (%) for categorical variables: the *p*-value was computed by a weighted chi-squared test.

### 3.2 Association between niacin intake and CKD in male participants

As shown in Table 2, weighted LRA indicated a significant association between niacin intake and CKD in male participants across Model 1 [OR: 0.973 (0.967–0.979)], Model 2 [OR: 0.991 (0.985–0.997)], and Model 3 [OR: 0.993 (0.987–0.999)]. Quartile 4 of niacin intake demonstrated a prominent association with the risk of CKD in Model 2 (Q1 vs. Q4, 0.752 [95% CI: 0.591–0.959], *p* < 0.05).

**Table 2:** Weighted LRA models of the association between niacin and CKD in males.

OR (95% CI)						
Participant	Model 1	*p*	Model 2	*p*	Model 3	*p*
Niacin (con)	0.973 (0.967 ~ 0.979)	<0.001	0.991 (0.985–0.997)	0.005	0.993 (0.987 ~ 0.999)	0.028
Niacin (cate)						
Q1 (≤21.483)	Reference		Reference		Reference	
Q2 (21.483 ~ 28.521)	0.703 (0.589 ~ 0.840)	<0.001	0.845 (0.700 ~ 1.019)	0.077	0.867 (0.718 ~ 1.046)	0.133
Q3 (28.521 ~ 37.098)	0.570 (0.479 ~ 0.677)	<0.001	0.817 (0.676 ~ 0.988)	0.037	0.859 (0.711 ~ 1.038)	0.115
Q4 (≥37.098)	0.383 (0.313 ~ 0.468)	<0.001	0.722 (0.579 ~ 0.899)	0.004	0.752 (0.591 ~ 0.959)	0.022
*p* for trend		<0.001		0.014		0.071

Model 1: Unadjusted model. Model 2: Adjusted for age, race, education level, and poverty-to-income ratio. Model 3: Additionally adjusted for smoking status, alcohol consumption status, BMI, hypertension, diabetes, and CVD.

For niacin quartiles, higher intake generally demonstrated a trend toward lower odds of CKD among male participants, particularly in Model 1 and Model 2. In Model 2, participants in Quartile 4 were associated with a 27.8% reduced risk of CKD compared to those in Quartile 1 (*p* < 0.05).

### 3.3 Analysis of RCS regression

**Figure 2:**
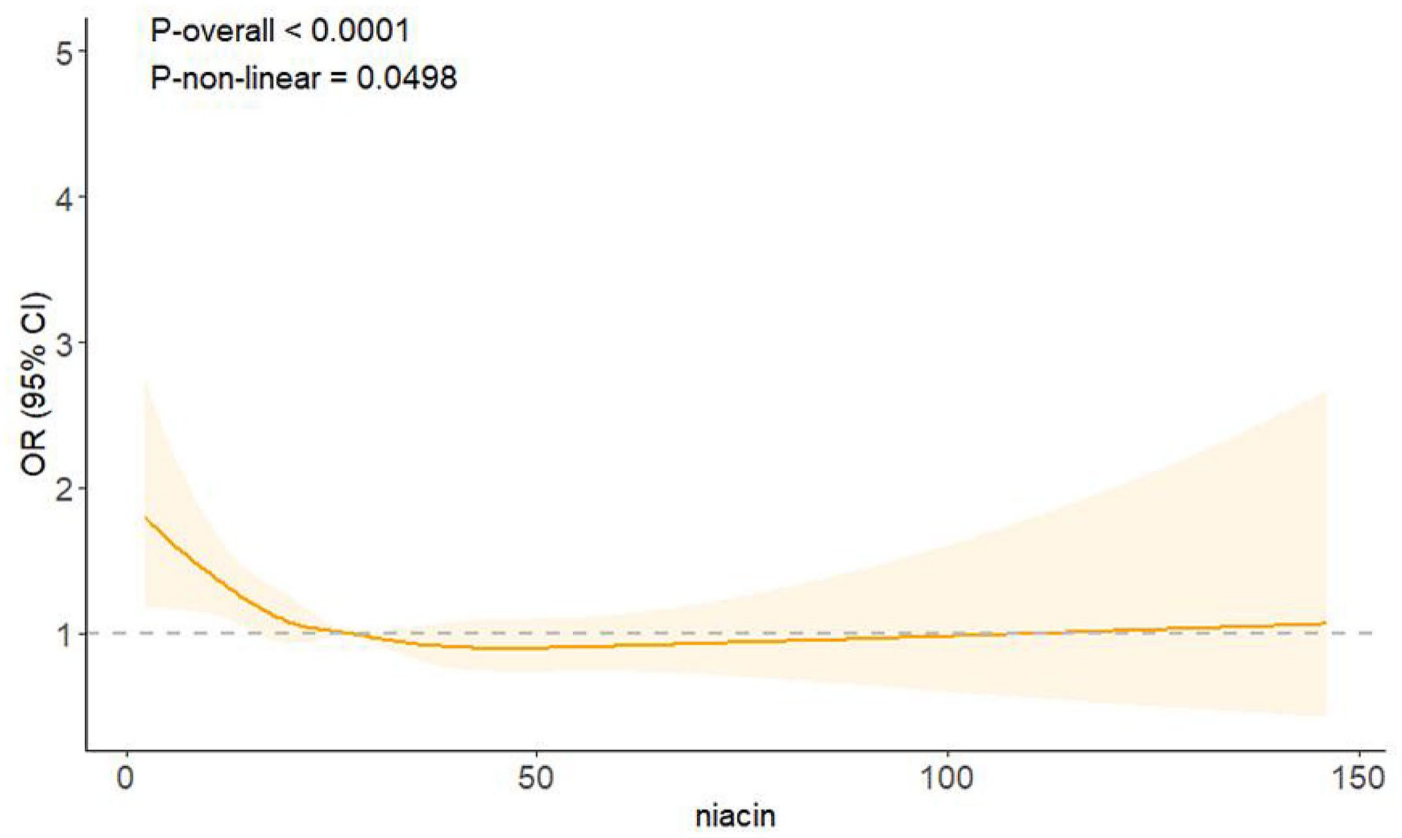
Association between niacin intake and CKD in male participants. Adjusted for age, race, education level, PIR, smoking status, alcohol consumption, BMI, hypertension, diabetes, and CVD. The solid line denotes the estimated values. The light color part denotes their corresponding 95% CIs.

RCS regression revealed an L-shaped association between niacin intake and CKD in male participants (Figure 2). A non-linear relationship between niacin intake and CKD in male participants was observed (*p*-non-linear < 0.05). The inflection point for niacin intake was 26.79 mg (*p* for log-likelihood ratio < 0.05) (Table 3). When niacin intake was <26.79 mg, a 1-unit increase in niacin was associated with a 2.2% reduction in the adjusted OR for CKD (OR: 0.978; 95% CI: 0.963, 0.994). When niacin intake was >26.79 mg, no significant association with CKD observed (OR: 0.999; 95% CI: 0.991, 1.008).

**Table 3:** Threshold effect analysis of niacin intake on CKD in male participants.

Participants	Adjusted OR (95% CI)	*p*-value
CKD		
Fitting by the standard linear model	0.993 (0.987 ~ 0.999)	0.031
Fitting by the two-piecewise linear model		
Inflection point	26.79 mg	
Niacin < 26.79 mg	0.978 (0.963 ~ 0.994)	0.006
Niacin ≥ 26.79 mg	0.999 (0.991 ~ 1.008)	0.821
*p* for log-likelihood ratio		<0.001

Adjusted for age, race, education level, PIR, smoking status, alcohol consumption, BMI, hypertension, diabetes, and CVD.

### 3.4 Stratified analyses

Further subgroup analysis unraveled that the connection between niacin intake and CKD in male individuals was not consistent (Table 4). Significant interactions were revealed between niacin intake and race, BMI, and CVD, indicating the association between niacin intake and CKD across these subgroups (*p* < 0.05). The association between niacin intake and CKD in male individuals was not statistically significantly different across strata, indicating that education level, PIR, smoking status, alcohol consumption, hypertension, and diabetes did not significantly influence this negative association (*p* for interaction > 0.05). Higher niacin intake (Quartile 4) was significantly associated with a reduced risk of CKD in the following subgroups: Mexican–American (OR: 0.510; 95% CI: 0.302, 0.861), non-Hispanic white (OR: 0.695; 95% CI: 0.504, 0.958), non-smokers (OR: 0.646; 95% CI: 0.432, 0.965), alcohol consumers (OR: 0.763; 95% CI: 0.587, 0.993), individuals with a BMI between 25 and 30 (OR: 0.631; 95% CI: 0.141, 0.961), those with a BMI of ≥ 30 (OR: 0.675; 95% CI: 0.468, 0.973), and individuals with diabetes (OR: 0.543; 95% CI: 0.357, 0.824).

**Table 4:** Subgroup analysis.

Subgroup	Q1 (≤21.483)	Q2 (21.483 ~ 28.521)	Q3 (28.521 ~ 37.098)	Q4 (≥37.098)	*p* for interaction
Race					0.015
Mexican–American	Ref.	0.668 (0.403 ~ 1.107)	0.686 (0.364 ~ 1.295)	0.510 (0.302 ~ 0.861)	
Other Hispanic	Ref.	0.663 (0.328 ~ 2.725)	1.272 (0.594 ~ 2.725)	0.834 (0.381 ~ 1.826)	
Non-Hispanic white	Ref.	0.813 (0.643 ~ 1.027)	0.829 (0.646 ~ 1.064)	0.695 (0.504 ~ 0.958)	
Non-Hispanic Black	Ref.	1.324 (0.960 ~ 1.826)	1.059 (0.714 ~ 1.570)	1.021 (0.682 ~ 1.529)	
Other races	Ref.	0.836 (0.440 ~ 1.589)	0.575 (0.308 ~ 1.074)	0.997 (0.456 ~ 2.177)	
Education					0.448
Under high school	Ref.	0.771 (0.555 ~ 1.072)	0.892 (0.560 ~ 1.421)	0.746 (0.468 ~ 1.188)	
High school	Ref.	0.980 (0.641 ~ 1.498)	0.803 (0.510 ~ 1.265)	0.730 (0.453 ~ 1.177)	
College	Ref.	0.853 (0.658 ~ 1.106)	0.881 (0.670 ~ 1.158)	0.775 (0.543 ~ 1.104)	
PIR					0.433
≤1.3	Ref.	0.734 (0.498 ~ 1.080)	0.811 (0.552 ~ 1.192)	0.745 (0.491 ~ 1.131)	
1.3–3.5	Ref.	1.018 (0.758 ~ 1.367)	0.866 (0.624 ~ 1.201)	0.834 (0.607 ~ 1.147)	
≥3.5	Ref.	0.770 (0.543 ~ 1.091)	0.826 (0.560 ~ 1.139)	0.675 (0.416 ~ 1.096)	
Smoke					0.560
Never	Ref.	0.718 (0.523 ~ 0.987)	0.745 (0.536 ~ 1.035)	0.646 (0.432 ~ 0.965)	
Former	Ref.	0.967 (0.717 ~ 1.304)	0.938 (0.687 ~ 1.281)	0.810 (0.532 ~ 1.232)	
Current	Ref.	1.020 (0.668 ~ 1.556)	1.008 (0.620 ~ 1.639)	0.891 (0.543 ~ 1.462)	
Alcohol					0.479
No	Ref.	0.875 (0.570 ~ 1.344)	1.127 (0.669 ~ 1.897)	0.646 (0.365 ~ 1.145)	
Yes	Ref.	0.866 (0.695 ~ 1.079)	0.824 (0.666 ~ 1.020)	0.763 (0.587 ~ 0.993)	
BMI					0.004
<25	Ref.	1.351 (0.881 ~ 2.071)	1.088 (0.684 ~ 1.730)	1.501 (0.943 ~ 2.392)	
25–30	Ref.	0.784 (0.541 ~ 1.135)	0.786 (0.546 ~ 1.132)	0.631 (0.141 ~ 0.961)	
≥30	Ref.	0.782 (0.591 ~ 1.035)	0.870 (0.625 ~ 1.209)	0.675 (0.468 ~ 0.973)	
Hypertension					0.803
No	Ref.	0.803 (0.452 ~ 1.425)	0.771 (0.511 ~ 1.161)	0.706 (0.442 ~ 1.129)	
Yes	Ref.	0.884 (0.727 ~ 1.074)	0.889 (0.712 ~ 1.111)	0.759 (0.570 ~ 1.010)	
Diabetes					0.179
No	Ref.	0.883 (0.685 ~ 1.139)	0.996 (0.787 ~ 1.260)	0.887 (0.674 ~ 1.167)	
Yes	Ref.	0.863 (0.623 ~ 1.198)	0.628 (0.459 ~ 0.861)	0.543 (0.357 ~ 0.824)	
CVD					0.041
No	Ref.	0.862 (0.687 ~ 1.082)	0.873 (0.694 ~ 1.097)	0.805 (0.616 ~ 1.052)	
Yes	Ref.	0.860 (0.583 ~ 1.268)	0.796 (0.554 ~ 1.142)	0.555 (0.308 ~ 1.001)	

### 3.5 Sensitivity analysis

After further adjustment for hyperuricemia, niacin intake remained significantly associated with a reduced risk of CKD [OR: 0.994, 95% CI: 0.988, 0.999] (Appendix Table 1).

## 4 Discussion

This cross-sectional study found that higher niacin intake was associated with a lower risk of CKD in male individuals. This association was not similar in subgroup analyses and interaction tests. A U-shaped relationship between niacin intake and CKD in male individuals was revealed. The inflection point was 26.79 mg, below which the higher niacin intake was significantly associated with a lower risk of CKD. In addition, niacin intake demonstrated significant interactions with race, BMI, and CVD in the presence of CKD.

Previous epidemiological studies have observed the association between niacin intake and kidney condition. A cross-sectional study in Mexico demonstrated that niacin intake was deficient for peritoneal dialysis patients under normal nutrition and malnutrition of any grade. Therefore, according to Dietary Reference Intakes, supplementing with niacin to compensate for losses during dialysis was recommended (20). A longitudinal study in the Netherlands revealed that niacin intake was a key factor in reducing mortality among kidney transplant recipients (21). Therefore, niacin intake appeared to be a significant indicator of CKD. A randomized controlled trial allocated 66 participants into the intervention and treatment control groups to investigate the impact of dietary status on CKD in clinical parameter outcomes. Although niacin intake in the intervention group was notably increased, CKD did not show a significant improvement (22), indicating no effect of nutritional supplements on CKD. The 3-month follow-up period may be too short, and a longer follow-up could potentially yield different results. Conversely, a cross-sectional study in Japan investigated the relationship between vitamin intake and CKD at the genetic level among the middle-aged population. It demonstrated a significant protective effect of niacin intake against CKD in the minor homozygotes of rs883484 (23). Bongarzone et al. used positron emission tomography to imagine niacin trafficking in rats, demonstrating a rapid radioactivity accumulation in the kidney, heart, eyes, and liver following the intravenous administration of [^11^C] niacin. In addition, niacin intake increased urine excretion in mice (24), indicating the benefit of niacin intake for CKD. Our findings suggest that high niacin intake has an association with a decreased risk of CKD.

Our study also identified an L-shaped association between niacin intake and CKD, with a breakpoint at 26.79 mg. A negative association was presented on the left side, but no association existed on the right side, implying a substantial threshold impact of niacin intake and CKD in male participants. According to the recommendation of the US Food and Nutrition Board, the upper limit of niacin intake was 35 mg daily (8). Long-term excessive niacin intake may increase the risk of adverse effects, such as liver toxicity and lipid metabolism disorders. This elevated intake could exacerbate CKD risk by potentially causing additional stress on the kidneys and interfering with metabolic homeostasis. When the niacin intake was less than 26.79 mg, it remained within the range of beneficial effects without causing harm. Several studies have also discussed the inflection point of niacin intake. From a cross-sectional study of migraine, the inflection point of niacin intake was 21 mg. When niacin intake was below 21 mg, a 1-unit increase in niacin intake was associated with a 2.5% risk of migraine (25). A study of depression indicated that the inflection point for niacin intake was 26.6 mg/day, revealing a negative association with depression when niacin intake was <26.6 mg/day and a positive association with depression when niacin intake was > 26.6 mg/day (26).

Based on the stratified analysis, we observed a protective effect of niacin intake (≥ 37.098) in male patients with CKD from Quartile 4, particularly among those with diabetes.

A cross-sectional study demonstrated that niacin intake had protective effects against diabetes when niacin intake was >15.01 mg (27). Another study revealed that high dietary niacin intake was positively associated with diabetes (28). Diabetes was a leading contributor to ESRD, heart disease, stroke, and liver cirrhosis. Diabetic kidney disease was also prevalent in individuals with type-2 diabetes (29). Therefore, sufficient niacin intake could protect against CKD, especially in those with diabetes. This finding highlighted the potential importance of dietary niacin intake in managing CKD risk in vulnerable populations. Further research is warranted to explore these associations and confirm the protective effects of niacin intake.

Our study revealed essential interactions between niacin intake and CKD risk across several subgroups, including race, BMI, and CVD status. These findings suggest that the association between niacin intake and CKD is inconsistent across all individuals. Specifically, higher niacin intake (Quartile 4) was significantly associated with a reduced risk of CKD in the following subgroups: Mexican–American individuals, non-Hispanic white individuals, and individuals with a BMI between 25 and 30 or over 30. This finding highlights that race and BMI are potential modifiers in the effects of niacin on CKD risk. For instance, in Mexican–Americans and non-Hispanic Whites, niacin intake was consistently associated with a lower risk of CKD, potentially reflecting racial/ethnic differences in metabolic pathways or dietary patterns. Similarly, individuals with a BMI between 25 and 30 and those with a BMI greater than 30 showed a significant reduction in CKD risk after higher niacin intake. This finding could be attributed to the fact that obesity is a known risk factor for CKD, and the potential metabolic and antioxidant effects of niacin may be particularly beneficial in this population. In addition, significant associations were observed in individuals with diabetes, a group that is at higher risk for developing CKD. Higher niacin intake in individuals with diabetes was associated with a reduced risk of CKD, suggesting that niacin might have protective effects against CKD in this vulnerable population. This finding aligns with that of previous studies, suggesting the potential benefits of niacin intake in managing kidney diseases in patients with diabetes. Furthermore, niacin deficiency may have implications beyond kidney disease, particularly in perioperative settings. Malnourished or frail patients are more susceptible to postoperative complications, and micronutrient deficiencies, including niacin, should be considered during preoperative assessment. A recent multicenter prospective study demonstrated the prognostic value of comprehensive malnutrition screening tools in surgical patients, underscoring the importance of nutritional status in perioperative outcomes (30).

This study has several advantages. First, the NHANES dataset employed a complex probability-based sampling design, and the sample size was sufficiently large to avoid selection bias. Second, the covariates used were suitable and enhanced the reliability of the conclusion. However, there are potential limitations. First, for the NHANES dataset, the questionnaire and interview are self-reported, and participants may provide inaccurate responses, possibly due to recall bias. The information bias will affect the measurement of niacin intake, potentially leading to overestimation or underestimation of the relationship between niacin intake and CKD in male participants. Second, the cross-sectional design limits the assessment of the causal relationship between niacin intake and CKD. Third, due to the limited clinical detail available in the NHANES dataset, we were unable to explicitly exclude participants with CKD caused by other etiologies such as renal autoimmune diseases (e.g., lupus nephritis), nephritic or nephrotic syndromes, or chronic kidney stones. These conditions may independently influence kidney function and act as confounding factors in our analysis. This limitation should be considered when interpreting the findings.

## 5 Conclusion

In conclusion, our study illustrates that dietary niacin intake is negatively associated with CKD risk in male participants. Diets rich in niacin content are essential for CKD management. Since niacin intake is a simple and feasible indicator of CKD in male participants, increasing niacin intake may be a cost-effective strategy to reduce CKD risk in male participants. However, larger longitudinal and interventional studies are needed to quantify the preventive and therapeutic benefits of dietary niacin intake for CKD management in clinical practice.

## Data Availability

The original contributions presented in the study are included in the article/Supplementary material, further inquiries can be directed to the corresponding author.
