# Supplementary material for: Association between niacin intake and chronic kidney disease in male participants—a cross-sectional study from the NHANES (2005–2018)
Source: Front Nutr. 2025 Jun 13;12:1578118. doi: 10.3389/fnut.2025.1578118 (PMC12202428; doi:10.3389/fnut.2025.1578118)
Supplement: Supplementary file 1 [file Table_1.docx]

**Appendix Table 1 Association between niacin and Odds rations (95% confidence intervals) of CKD in males, NHANES 2005-2018**

| **Participant** | **Model 3** | **p** | **Model 4** | **p** |
| --- | --- | --- | --- | --- |
| **Niacin (con)** | 0.993 (0.987~0.999) | 0.028 | 0.994 (0.988~0.999) | 0.019 |
| **Niacin (cate)** |  |  |  |  |
| Q1 (≤21.483) | Ref. |  | Ref. |  |
| Q2 (21.483~28.521)) | 0.867 (0.718~1.046) | 0.133 | 0.981 (0.865~1.112) | 0.076 |
| Q3 (28.521~37.098) | 0.859 (0.711~1.038) | 0.115 | 0.891 (0.771~1.029) | 0.114 |
| Q4 (≥37.098) | 0.752 (0.591~0.959) | 0.022 | 0.830 (0.707~0.974) | 0.023 |
| **p for trend** |  | 0.071 |  | 0.103 |
| Model 3: Adjusted for age, race, education level, poverty-income ratio Smoking status, Alcohol status, BMI, Hypertension, Diabetes, and CVD. Model 4: Adjusted for age, race, education level, poverty-income ratio Smoking status, Alcohol status, BMI, Hypertension, Diabetes, CVD, and Hyperuricemia. | | | | |
